# Empyema thoracis: Surgical management in children

**DOI:** 10.4103/0971-9261.57698

**Published:** 2009

**Authors:** Prema Menon, Ravi Prakash Kanojia, K. L. N. Rao

**Affiliations:** Department of Pediatric Surgery, Advanced Pediatric Center, Postgraduate Institute of Medical Education and Research, Chandigarh, India

**Keywords:** Chest tube drainage, empyema thoracis, fibrinolytic agents, open decortications, thoracocentesis, video-assisted thoracoscopic surgery

## Abstract

Empyema thoracis can produce significant morbidity in children if inadequately treated. Correct evaluation of the stage of the disease, the clinical condition of the child and proper assessment of the response to conservative treatment is crucial in deciding the mode of further surgical intervention. This ranges from intercostal chest tube drainage and video-assisted thoracoscopic surgery to open decortication. Surgical decortication becomes mandatory in neglected cases; it gives very gratifying results ameliorating the disease rapidly and is well tolerated by young patients. This article reviews the current literature and discusses the important considerations while managing these patients. Indications for surgery are highlighted, based on our large experience at a tertiary care center.

## INTRODUCTION

Empyema, an accumulation of infected fluid within the thoracic cavity, is commonly secondary to post-infectious pneumonia. It can also occur after thoracic operations, trauma or esophageal leaks. The American Thoracic Society has described 3 stages of empyema, namely exudative, fibrinopurulent and organized, more or less based on the characteristics of the contents of the pleural cavity.[1]

Apart from the fluid, organized fibrinous deposits appear early in the disease preventing complete drainage of fluid as well as penetration of antibiotics. An inflammatory peel of variable thickness soon forms preventing complete lung expansion. This leads to a variable clinical course creating a lot of confusion about the exact method of management. The disease becomes chronic and delayed referral to the surgeons by the community physicians is common world over. We have also observed like others, a definite discrepancy in the treatment modality advocated by non-surgical and surgical specialists.[2] Surgeons themselves are reluctant to operate partly due to inexperience and also because of the fear of “post-operative morbidity” mentioned in pediatric literature. However, it is a condition which if approached by the correct surgical technique gives excellent results with minimal morbidity.

Empyema usually presents with persistent high grade fever, cough, tachypnea / dyspnea, irritability and chest deformity. Malnourishment and wasting are associated. Our experience has been that several consultations have been taken over the previous 3-6 weeks and the child being administered various broad spectrum antibiotics including empirical anti-tuberculous treatment. Rarely the empyema may be bilateral. Here the duration of symptoms is usually longer. There is associated anemia and other secondary hematological abnormalities

Often, there is a history of multiple chest tube insertions. After an initial period of response, the drainage has usually ceased with no commensurate improvement in the clinical condition of the patient.[3] Referral is usually with multiple chest radiographs, hemograms and culture reports but the most important investigation is a recent contrast enhanced computed tomographic (CECT) scan of the chest. This and the clinical status of the child are the cornerstones for reaching the correct decision on surgical management.

## EVALUATION OF THE CHILD WITH SUSPECTED EMPYEMA

**Figure 1:**
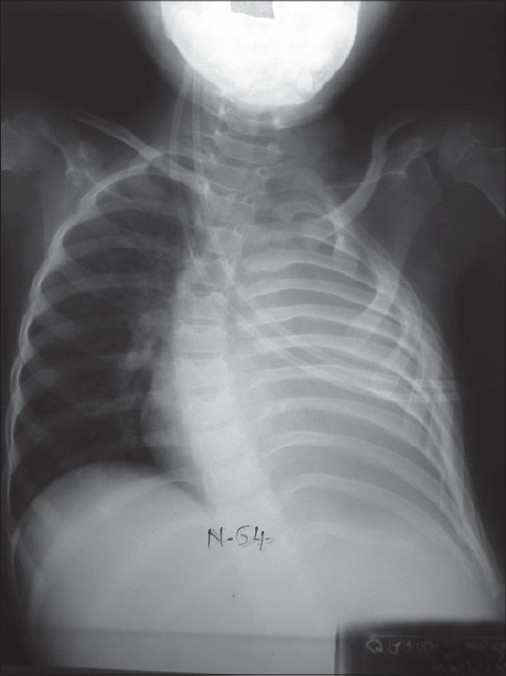
Chest X-ray PA view showing complete opacity on left side and scoliosis

The following investigations are performed: complete hemogram, coagulation profile and serum albumin. A plain X-ray chest may show opacity, loculated air, pleural peel and scoliosis [Figure 1]. Loculated pus and pulmonary entrapment are not easily made out. Ramnath *et al*, devised an ultrasound based grading system subdividing patients into low grade or high grade categorized by presence or absence of septations, fronds (like a leaf with many divisions) and loculi. Those with higher grade uniformly did better on surgical intervention with lesser hospital stay.[4]

In our own experience, we have found that sonography is unable to accurately differentiate between patients in stage 2 and stage 3 leading to wastage of time in conservative treatment.

Needle thoracocentesis is then performed to confirm the diagnosis of suppurative pleural effusion and get a specimen for culture and sensitivity to antibiotics. The colour and consistency are noted and the p^H^, glucose levels and lactic dehydrogenase (LDH) analyzed. In patients who have presented early, i.e. within 1 week of onset of symptoms these investigations are sufficient. However those presenting later require a CECT scan.

## IMPORTANCE OF CECT SCANS OF CHEST

Computed tomographic scan with contrast enhancement should be performed with lung and mediastinal windows to reveal the exact extent and nature of the disease. Very few authors have realized the importance of CECT chest while deciding for surgery.[5] The majority of studies that we reviewed were based on a chest radiograph and not a CT scan leading to incorrect judgment of the stage of the disease as well as delay in surgical intervention with consequent increased morbidity.[2,6]

**Figure 2:**
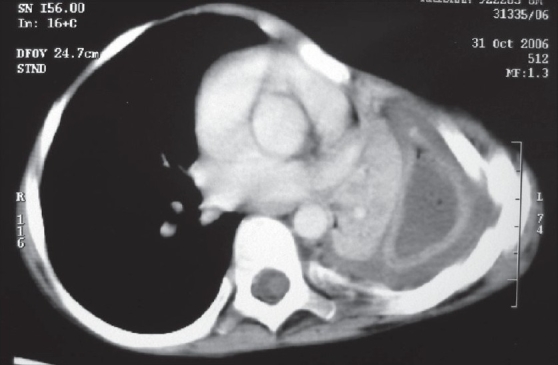
CECT scan of same patient as in Fig. 1: Mediastinal window showing crowding of ribs, collapsed and entrapped left lung and pleural space full of debris

**Figure 3:**
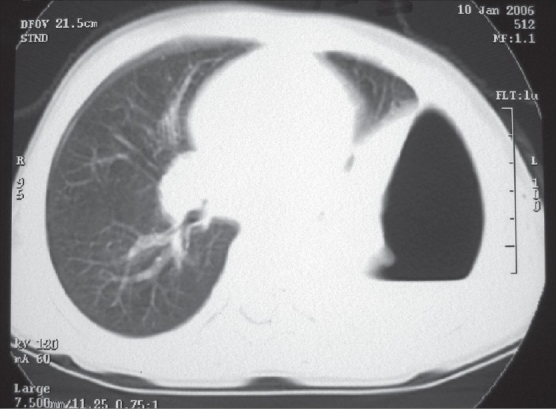
CECT, lung window: Normal right lung, loculated air and pus posteriorly and normal lung anteriorly in left hemithorax

**Figure 4:**
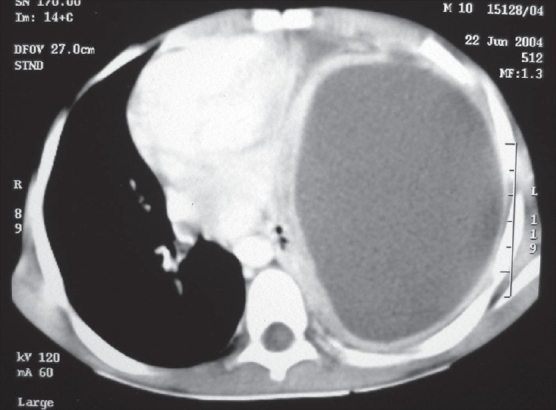
CECT, mediastinal window: Large collection in entire left hemithorax

A chest radiograph provides only two dimensional information. It may only show opacity occupying a certain area of the hemithorax, which may be secondary to consolidated parenchyma, pleural peel or a lung abscess. On the other hand, the ability of CECT to show the thorax in various sections and planes helps to reveal precise information about the location, density and volume of the fluid along with the thickness of the pleural peel and the status of the underlying lung with its degree of entrapment [Figure 2]. Loculations may be single or multiple, may contain pus, air or both [Figure 3]. Sometimes, the loculated pus can occupy the entire hemithorax and masquerade as a lung cyst [Figure 4].

**Figure 5:**
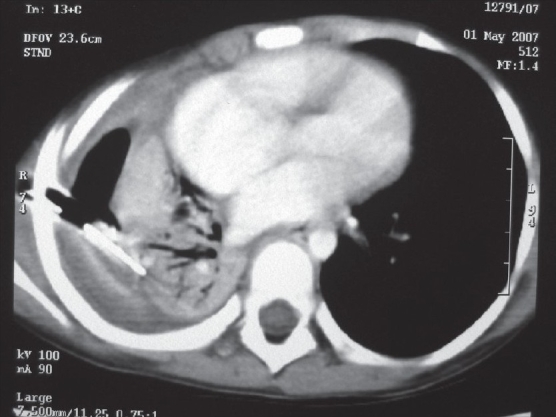
CECT, mediastinal window: Chest tube missing the pocket of air and entering lung tissue

A common clinical problem encountered is that the chest tube has stopped draining although the patient is still symptomatic. This may be secondary to the tube missing the pocket of loculated air or pus altogether or due to the consistency of the debris [Figure 5]. Lung expansion may be prevented by the thick pleura encasing the lung or presence of necrotic lung tissue. These features are easily made out on a CT scan as compared to a chest radiograph.

**Figure 6:**
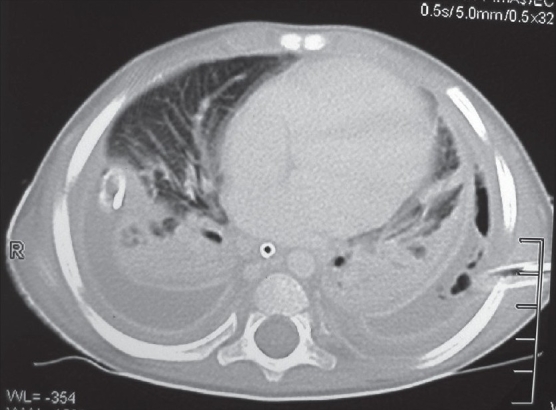
CECT, mediastinal window: Bilateral empyema thoracis

**Figure 7:**
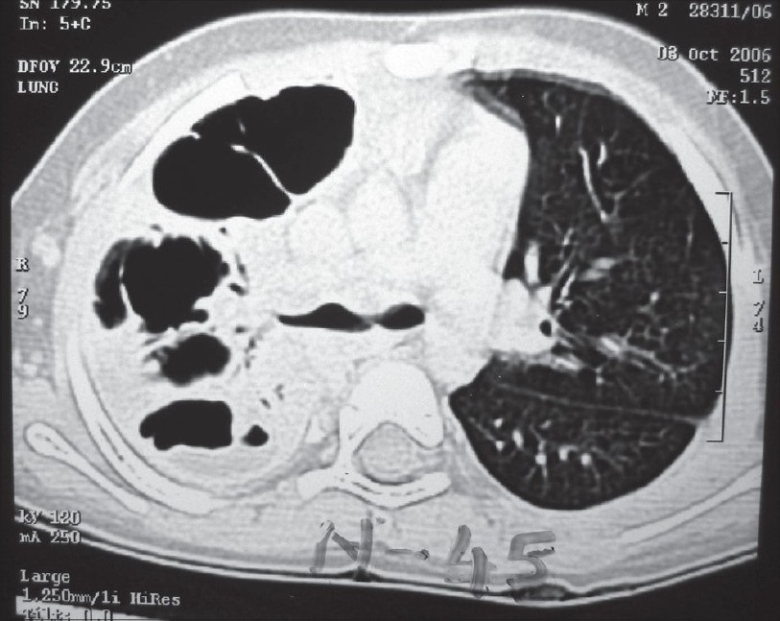
CECT, lung window: Loss of lung architecture, multiple thin walled cysts and decreased enhancement consistent with cavitary necrosis of right lung

Patients may present with bilateral empyema [Figure 6]. Lung necrosis may be associated in any of the scenarios. It may be secondary to incorrect placement of the chest tube. More often it is due to the severity of the disease process and may involve a part of a lobe, the entire lobe or rarely the entire lung [Figure 7].

In a study from Taiwan, the radiographic manifestations associated with severity of disease included bilateral involvement, thickness of empyema > 3 cm mantle by chest CT scan in lying position, multiple loculated effusions, extent of empyema > 1/3 hemi thorax and presence of air-fluid level in CT scans.[7] Hoff *et al*, in 1989 gave an empyema severity score (ESS) to ascertain patients who may progress to a more severe disease based on the pleural fluid characteristics, chest radiography findings and type of infection.[8] Although the pleural fluid investigations (fluid with pH < 7.2, glucose < 40 mg/dL, LDH >1000 IU/dL, protein > 2.5 g/dL, WBC > 500/*μ*L, and specific gravity greater than 1.018) are good markers, this score singularly suffers from basing the severity on a chest radiograph alone and not a CT scan.

Evidence of persistently collapsed lung requires surgical intervention. In a symptomatic patient, based on serial chest X-rays and CECT of chest, our indications for thoracotomy and decortication are as follows:

1. Thick pleural peel encasing the lung [Figure 8]

**Figure 8:**
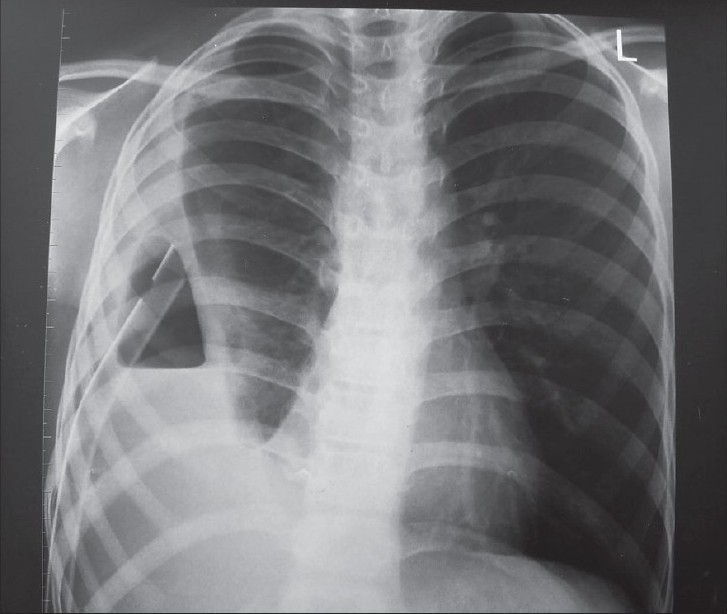
Chest X-ray: Thick pleural peel not allowing full expansion of the right lung
2. Pleural debris
  - >20% volume
  - <20% volume but symptoms (fever/tachypnea) are persistent
3. Multiloculated empyema [Figure 9]

**Figure 9:**
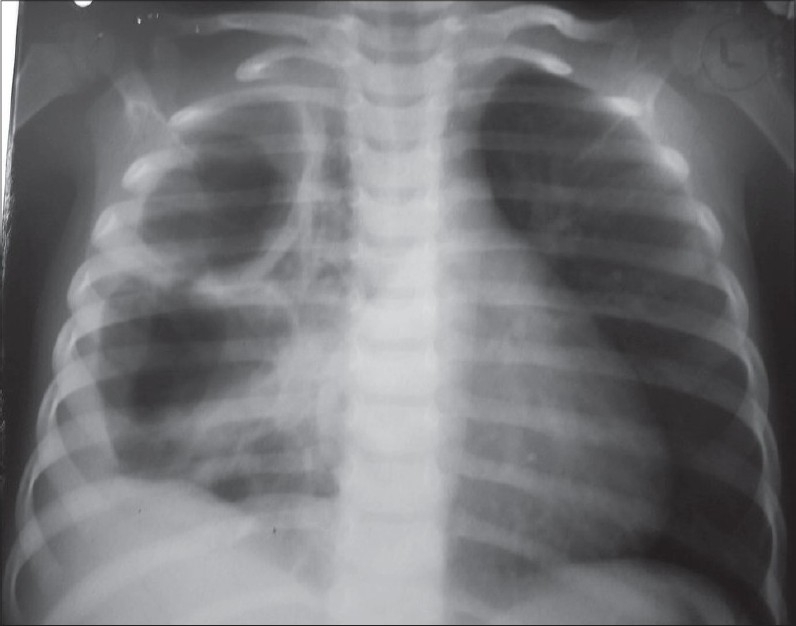
Chest X-ray showing multilple loculations in right hemithorax
4. Cavitary lung necrosis [Figure 7]
5. Persistent broncho-pleural fistula with collapse of lung.

## MANAGEMENT BASED ON THE STAGE OF THE DISEASE

All patients are started on intravenous (i.v.) fluids, i.v. broad spectrum antibiotics and oxygen inhalation. Some may require ventilatory support. Further treatment would be based on the stage of the disease.

### Stage 1 (Exudative phase)

The fluid is usually thin with free communication in the thoracic cavity. It may be serous or cloudy but is generally sterile. There is good response to antibiotic therapy in most with some requiring an additional paracentesis or a short period of chest tube drainage. These children do not require surgical intervention.

Traditionally, use of only antibiotic therapy with or without closed chest tube drainage has been popular among pediatricians.[9] This is partly due to the belief that children have a greater capacity to resorb thickened pleura as compared to adults.[6] Closed tube thoracostomy is a primary surgical maneuver but is usually performed by pediatricians who have taken the decision to insert the chest tube, only on the basis of opacity seen on a plain chest radiograph (postero-anterior view). This often leads to the chest tube being placed too anteriorly or entering into lung tissue. Some of the problems of blind catheter placement can be avoided by image-guided catheter placement. Chest sonography has been found to be useful by some authors to delineate the loculi and locate the site for aspiration and insertion of chest tube in the correct intercostal space.[6,10]

In a study from Phillipines over a 14 year period, 31 children managed only by tube thoracostomy were studied. Only 6% achieved lung reexpansion a week later. Sixty four percent did not achieve lung reexpansion even after 3 weeks and most had prolonged hospital stay.[11] Patients should therefore always be monitored for features of persistent sepsis while on conservative treatment. This includes high grade fever, loss of appetite, loss of weight, irritability as well as reduced lung capacity manifested by tachypnea and dyspnea. This warrants an early surgical consultation.[12]

### Stage 2 (Fibrinopurulent stage)

This lasts 7-10 days and is associated with fibrinous debris. The fluid is thicker, opaque and cultures are positive.

In a small study from Brighton, U.K., 14 patients were studied.[6] The authors grouped them as stage 2 based on the colour of the pleural fluid, chest X-ray and loculation on ultrasound. Patients had fever before admission for 8 days (median). Chest tubes were placed soon after admission under short general anesthesia after ultrasound imaging, which included a finger sweep by the pediatric surgeon during insertion. Intravenous antibiotics at high doses were given for 5-23 days (median 10 days). Resolution of fever and constitutional symptoms were noted 1-7 days after chest tube insertion. At discharge patients continued to have marked opacity of more than half of the hemi thorax. Chest tubes often remained in situ even though not draining because of opacity seen on the chest X-ray. Radiological resolution again based entirely on chest X-ray took 2-16 months to resolve. These problems of groping in the dark could have been done away with if a better method; i.e. CT scan had been used by the authors. The paper also does not mention whether these patients continued to receive oral antibiotics at discharge given that opacity was still seen on chest X-ray.

In a study by Chan *et al*[2] at the Montreal Childrens Hospital, 39 of 47 children with Empyema were in stage 2. Only 7 had complete drainage with thoracostomy alone. Another 7 (18%) did not respond to antibiotics and thoracostomy drainage and underwent decortication. The remaining 25 had incomplete drainage of loculations with prolonged hospital stay. Of these, 7 ultimately required decortication.

Often due to multiple loculations, symptoms persist and patients require more than one chest tube insertion. To circumvent this problem, since the 1990's, 2 new treatment modalities have been described; fibrinolysis which can promote pleural drainage and circulation and early video assisted thoracoscopic surgery (VATS)[13] Following the rapid popularity of VATS amongst surgeons, a criticism that came up was that children who may have responded to less invasive therapies were being subjected to surgery and anesthesia. Because of active lung infection these patients may be at a higher anesthetic risk while being in a catabolic phase.[14]

### (A) Role of fibrinolytic agents

In theory, patients with loculations and septations should respond to fibrinolytic agents, thereby reducing the hospital stay instead of being treated only with chest tube drainage.[15] However, solid evidence for the role of agents like urokinase and streptokinase is lacking. There are also concerns regarding fibrinolytic therapy including bleeding and development of bronchopleural fistula.

Gates *et al*,[14] reviewed patients with para-pneumonic effusions treated by different methods over a 2 year period. Patients were studied in 4 groups, receiving chest tube alone; chest tube and fibrinolytics; chest tube, fibrinolytics and surgery or surgery alone. Out of 54 patients, 6 underwent surgery initially followed by another 5 after failure of fibrinolytics and tube drainage. The agent used was altepase and the authors observed that response would be seen, if at all, within 36-48 hours. There was no difference in the duration of hospital stay whether fibrinolytics were given immediately after chest tube insertion or 48 hours later. Although the authors showed good response to the agent, the groups were not comparable as the characteristics of the pleural fluid glucose and LDH were different in the various groups as were the sonographic features. A positive feature of the study was that a majority of the tubes were placed by interventional radiology and ultrasonography was performed in all cases.

In a Liverpool study on 38 children in a 3 year study period, the final outcome after a small tube thoracostomy with intrapleural urokinase was shown to be similar to that of thoracoscopy or thoracotomy. However, children who underwent thoracotomy required no further procedure and also had amelioration of pyrexia more rapidly.[16] Balci *et al*, using urokinase in 28 patients with loculated empyema compared it with another 43 patients who had undergone thoracotomy and decortication.[17] In the former group, treatment was ineffective in 21%, partial resolution was seen in 10.7% and one died of sepsis and pleural hemorrhage. All the patients who underwent surgery recovered completely. Only one randomized trial compared intrapleural urokinase in 60 children vs. saline. However there were doubts whether the patients truly had fibrinopurulent empyema.[18]

In fact, in one study on complicated para-pneumonic effusions, the percentage of patients requiring surgery was higher in those who had intrapleural streptokinase than a comparative group which had only simple closed chest tube drainage. There was no improvement in the duration of fever, chest tube drainage or hospital stay.[19]

The results of the First Multicenter Intrapleural Sepsis Trial (MIST 1) are worth reading.[20] This was a double blind randomized trial performed in 52 centres in the United Kingdom. There were a total of 454 patients who were assigned to receive either 250,000 IU of intrapleural streptokinase twice daily for 3 days or a placebo. Inclusion criteria included purulent pleural fluid positive on culture and with a pH below 7.2 apart from clinical indicators of infection. Patients received treatment as per routine care. This study comprehensively showed that intrapleural administration of streptokinase did not improve mortality, rate of surgery, radiographic outcome or the length of hospital stay and in fact made the comment that it should be avoided in pleural infection. In addition, serious adverse events like chest pain, fever or allergy were more common in the streptokinase group. Although this was not based on children, the study can be easily interpolated to the pediatric age group because of its statistical significance. The authors concluded that the reason for failure was that streptokinase only breaches the barrier between pockets of pus. The fluid being viscous and lumpy is unable to drain through the chest tube. The authors therefore suggest that deoxyribonuclease (DNase) may be a better agent as it reduces viscosity. However this important and recent paper again falls into the same pitfall committed by previous studies by basing itself only on a chest radiograph without knowledge of correct situation on a CECT chest.

**(B) Video assisted thoracoscopic surgery** and decortication is the treatment of choice nowadays in stage 2. Early use of VATS decreases the number of procedures and studies performed as well as the duration of chest tube drainage.[21] It allows breakage of fibrinous strands through a small incision and complete evacuation of pus with thorough irrigation. Compared to a thoracotomy, there is reduced pain and hospital stay, less morbidity and better cosmesis.

In our experience, patients do not get referred to the surgeon at an early stage making thoracoscopic debridement extremely difficult. Due to delayed stage, bleeding secondary to the inflammation is common making endoscopic visibility poor and consequent conversion to open procedure. A study conducted in Switzerland had similar observations.[22] Over an 18-month period, 9 children came up for surgery after initial chest tube drainage. In 5 children, where the duration of illness was less than 14 days, thoracoscopy was performed. In all these patients, it failed to completely clear the disease and formal thoracotomy with decortication had to be performed.

In a study conducted from 1990 to 2006 in a tertiary referral centre in Scotland, among 28 children with empyema, 14 required surgeries. While 8 underwent open decortication, of the 6 who initially had thoracoscopic decortication 2 had to be converted to open procedure. This has to be kept in mind that the primary aim is not only to remove pus but also facilitate complete expansion of the lung by removing the visceral pleural peel. This may not be always achievable by thoracoscopic approach due to the thickness of the peel.[23]

Numerous approaches have been described including placement of 1, 2 or 3 ports as per imaging studies. One-lung ventilation has been described either with the aid of a double lumen endotracheal tube or main stem intubation but is rarely necessary. The patient is placed in a lateral decubitus position over an axillary role. Based on the intercostal space and the age of the child, 3 or 5 mm ports are inserted. CO_2_ insufflation pressures are kept low at around 4 mm Hg which helps in slow disruption of pleural adhesions and lung collapse. Coagulum and interlobar collections are removed. Adherent peel is removed completely from the pleural surfaces. As with open surgical techniques following decortication, the lung should be inflated and seen to occupy the entire hemi thorax. Otherwise it can predispose to persistent atelectasis and recurrent empyema. The pleural space is irrigated with antibiotic solution and a chest tube placed via a port site. In difficult situations, conventional instruments can be inserted through port sites. Angled telescopes, 3 and 5 mm instruments, gentle technique and experienced operating team are essential.

Shah *et al*, published their experience with thoracoscopy in 10 patients.[24] Mild fever was present in 3 cases and 4 patients had tachypnea. Multiple loculations were seen on thoracoscopy only in 2 patients. Eight cases were said to be in fibrinopurulent phase. Of the 10, 3 were converted to open surgery, with one in the organized phase and another patient requiring a pneumonectomy for destroyed lung. The only complication encountered by them postoperatively was a small pneumothorax which resolved spontaneously. Patients stayed in hospital after surgery for 7-10 days.

In a retrospective analysis of children below 18 years in a primary and tertiary referral centre for children in Taiwan, early VATS was compared to salvage VATS.[7] The cutoff for early and salvage VATS (in symptomatic patients) was taken as 7 days of conservative treatment. Children who had received parenteral antibiotics for more than 5 days or those who had a thoracostomy tube insertion prior to admission were excluded from the study. The mean duration of hospitalization was shorter in patients having early VATS as compared to salvage VATS (mean 18 vs. 28 days). The authors concluded that early VATS should be adopted not later than 7 days after failure of appropriate antibiotic therapy and adequate drainage of empyema. A Cochrane database study of the period 2001-2005 which included children and adults concluded that although there were no proper randomized studies, VATS had a significantly higher success rate and patients spent lesser time in hospital compared to chest tube drainage with streptokinase.[25]

### Stage 3 (Organized stage)

In this, the organized stage, in addition to loculated pus or air, there is marked thickening of the pleura with encasement of the lung. These are better delineated on a CT scan as opposed to a chest radiograph. The gold standard for surgical treatment at this stage remains thoracotomy and decortication. Majority of the patients attending the tertiary care centers like ours, are in this stage of the disease with massive debris, loculations or thick leathery peel encasing the lung and open surgical decortication becomes mandatory. Even if the child is on a ventilator requiring respiratory assistance, many in our setup have benefited from open decortications. They could be weaned off the ventilator easily [Figure 6].

#### Goals of Surgery

(1) Thorough pleural debridement (2) release of encased lung parenchyma by carefully removing the thick plural peel from the entire lung surface and making the lung expand (3) meticulous closure of all major air leaks and (4) excision of necrotic lung tissue, which may be required in nonresponsive necrotizing pneumonias, fungal pneumonias and parenchymal abscesses.

**Figure 10:**
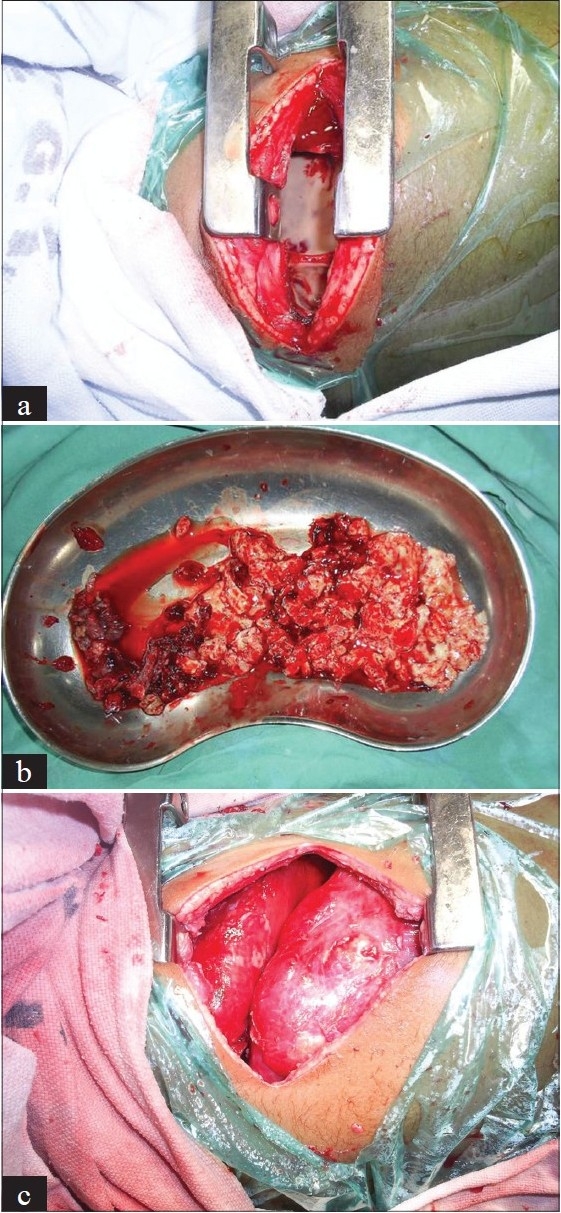
Open thoracotomy with decortication. (a) Appearance on opening the thorax: non visible lung, thick pleura and debris. (b) Large quantity of pleural debris. (c) After meticulous debridement and decortication, full expansion of the lung may be seen

Only standard open thoracotomy can attain all the above described 4 surgical goals in comparison to thoracoscopic procedures and the minithoracotomy described by Raffensperger[26] [Figure 10a–c]. In addition, lung expansion can be easily assessed prior to closure.

#### Procedure for open thoracotomy and decortication

The patient is placed laterally with the affected side up and the intercostal spaces increased by bolster under the chest and the arm lifted up. A posterolateral thoracotomy is performed without excision of the rib through the 4^th^ or 5^th^ intercostal space. Often the intercostal space is considerably narrowed out but access is gradually gained by retraction. The pleural space is entered and pus samples taken for culture and sensitivity. Pieces of pleura are taken for histopathological examination especially to rule out tuberculosis. Loculi are broken and the entire pleural space thoroughly debrided. The thick pleural peel is carefully and meticulously removed from the surface of the entire lung releasing the encased lung. Lung expansion should be assessed by asking the anesthetist for manual ventilation. All significant air leaks are meticulously closed using vicryl (absorbable) sutures. These maneuvers are not easy to perform with thoracoscopy in the confined space in the presence of inflamed tissue. It is very important to thoroughly irrigate the chest cavity with large amounts of normal saline until all flakes are cleared. One or two chest tubes of large bore are inserted which should be well fixed. An intercostal local anesthesia block is given and after approximating the ribs, the chest is closed. The chest tubes should not be clamped during transfer to the ward; otherwise it can predispose to subcutaneous emphysema.

Lung necrosis and lung abscesses should be dealt with during the same operative procedure or else it will prolong the postoperative course considerably with persistent air leak and fever. Some patients may require lobectomy and rarely pneumonectomy. It is often associated with bronchopleural fistula where there is a long history of persistent major air leak through the chest tube. Apart from thorough debridement with removal of pus, debris and visceral pleura, surgical treatment involves excision of necrotic lung and closure of involved airway in a healthy area.

#### Post-operative care

This includes continued antibiotic cover, good analgesia and care of the intercostal chest tube drain (ICTD). A chest x-ray is taken the evening of the surgery to confirm adequate lung expansion and correct position of the ICTD. The ICTD should be removed only when the output is nil and the chest radiograph shows good lung expansion. It should be milked frequently and care should be taken that it does not get blocked. Patients should be given frequent steam inhalation, chest physiotherapy and encouraged to perform incentive spirometry. The postoperative stay is usually uneventful and requirement for ventilatory care is extremely rare. Children recover fast and results are gratifying.

A few authors have gone for early decortication. In a study from Turkey, Gun *et al*, reviewed 79 cases of empyema over a 15 year period.[5] Except 3 patients, all received antibiotics and chest tube drainage. Patients underwent decortication at the end of 10 days if there was persistent fever, dyspnea, air leak or lack of resolution on CT scan. They were discharged on the 5^th^-8^th^ postoperative day. Quick resolution of symptoms after decortication has been our experience too. A study from Liverpool of 47 children in 3 consecutive 6 year periods from 1980-97, showed a positive shift in management in their institute towards early thoracotomy due to associated prompt symptomatic recovery.[27] Conservative management was seen to result in prolonged fever and recurrent effusions in 20% cases leading to prolonged hospital stay. Sixteen of 20 (80%) children initially treated with tube drainage required thoracotomy and debridement or decortication. Major complications were noted in 7 who had significantly delayed thoracotomy which included recurrent empyema with lung abscess, scoliosis, restrictive lung disease, bronchopleural fistula and sympathetic pericardial effusion.

In Hoff *et al*, study, in 18 of the 51 patients of empyema who underwent decortication, no deaths or morbidity were associated.[9] No complications were observed in other studies as well.[2,24] Scoliosis resolved after completion of treatment in all 4 patients in whom it was noted in the Montreal study.[2]

## CONCLUSIONS

Empyema thoracis following parapneumonic effusions is a progressive disease and associated with a lot of morbidity unless treated adequately and on time. In patients who present within a week of onset of fluid collection, antibiotics would be sufficient. The pleural fluid should be sent for culture and analyzed for purpose of prognostication. If the period is more than 7 days, then an additional CECT chest should be performed which exactly quantifies the disease and the patient treated as per stage and progress of disease process. Chest X-ray is only suitable for monitoring of lung expansion and relying solely on it delays decision making as it is unable to reveal the disease severity and predict the need for surgical intervention. CECT chest is necessary for complete evaluation of a child with suspected empyema, especially for surgical decision making. Early surgery reduces morbidity and hospital stay. Decortication in empyema is safe, effective and well tolerated by children.
